# Long non‐coding RNAs: How to regulate the metastasis of non–small‐cell lung cancer

**DOI:** 10.1111/jcmm.15054

**Published:** 2020-02-12

**Authors:** Cheng Fang, Lixin Wang, Chenyuan Gong, Wenbin Wu, Chao Yao, Shiguo Zhu

**Affiliations:** ^1^ Center for Traditional Chinese Medicine and Immunology Research Shanghai University of Traditional Chinese Medicine Shanghai China; ^2^ Department of Immunology and Pathogenic Biology School of Basic Medical Sciences Shanghai University of Traditional Chinese Medicine Shanghai China; ^3^ Laboratory of Integrative Medicine School of Basic Medical Sciences Shanghai University of Traditional Chinese Medicine Shanghai China; ^4^ Experiment Animal Center Experiment Center for Science and Technology Shanghai University of Traditional Chinese Medicine Shanghai China

**Keywords:** cancer stem cell, epithelial‐mesenchymal transition, long non‐coding RNAs, metastasis, non‐small‐cell lung cancer

## Abstract

Non–small‐cell lung cancer (NSCLC) has become the most lethal human cancer because of the high rate of metastasis. Hence, clarifying the molecular mechanism underlying NSCLC metastasis is very important to improve the prognosis of patients with NSCLC. Long non‐coding RNAs (LncRNAs) are a class of RNA molecules longer than 200 nucleotides, which can participate in diverse biological processes. About 18% of human LncRNAs were recently found to be associated with tumours. Many studies indicated that aberrant expression of LncRNAs played key roles in the progression and metastasis of NSCLC. According to the function in tumours, LncRNAs can be divided into two classes: oncogenic LncRNAs and tumour‐suppressor LncRNAs. In this review, we summarized the main molecular mechanism of LncRNAs regulating NSCLC metastasis, including three aspects: (a) LncRNAs interact with miRNAs as ceRNAs; (b) LncRNAs bind with target proteins; and (c) LncRNAs participate in the transduction of different signal pathways. Then, LncRNAs can exert their function to regulate the metastasis of NSCLC through influencing the progression of epithelial‐mesenchymal transition **(**EMT) and the properties of cancer stem cell (CSC). But, it is necessary to do some further research to demonstrate the LncRNAs particular regulatory mechanism of inhibiting the metastasis of NSCLC and explore new drugs targeting LncRNAs.

## 1 INTRODUCTION

The lung cancer is the leading cause of cancer‐related death in China and is responsible for more than 1 million deaths around the world annually.^1^, ^2^ Despite there are many methods to treat the lung cancer cases, the overall survival is still poor because patients were at advanced stages when diagnosed.^3^ Non–small‐cell lung cancer (NSCLC), including adenocarcinoma, squamous cell carcinoma and large cell carcinoma, composes about 85% of lung cancers, and more than half of patients with newly diagnosed NSCLC have metastatic disease.^4^ Because of the high rate of metastasis, NSCLC has become the most lethal human cancer.^5^ Thus, understanding the molecular basis underlying NSCLC progression and metastasis is important to improve the treatment and prognosis of patients with NSCLC.

Long non‐coding RNAs (LncRNAs) are a class of RNA molecules longer than 200 nucleotides, which can participate in diverse biological processes, including cell differentiation, modulation of apoptosis and invasion, reprogramming stem cell pluripotency and parental imprinting.^6^ The main characteristics of LncRNAs are that these RNAs are able to be transcripted, but cannot be translated into proteins, so they exert their respective biological functions at RNA level.^7^ Meanwhile, their abnormal expression is closely related to a variety of diseases, especially tumours.^8^ About 18% of human LncRNAs were recently found to be associated with tumours.^9^ Many studies demonstrated aberrant expression of LncRNAs played key roles in the progression and metastasis of NSCLC.^10^, ^11^, ^12^ According to the function in tumours, LncRNAs can be divided into two classes: oncogenic LncRNAs and tumour‐suppressor LncRNAs. Nowadays, LncRNAs have become a new therapeutic target for treating NSCLC metastasis.

More and more evidence indicated that LncRNAs were involved in tumour invasion and metastasis. Hence, our main purpose is to review recent literature on the relationship between LncRNAs and the metastasis of NSCLC.

## 2 MAIN MOLECULAR MECHANISM OF LncRNA TO REGULATE THE METASTASIS OF NSCLC

### 2.1 LncRNAs interact with miRNAs as ceRNAs

Many researchers have proved that LncRNAs could regulate the level of miRNAs, which in turn regulate the expressions of miRNA's target genes. LncRNAs might compete with miRNAs as miRNA sponges in tumour progression. Competing endogenous RNA (ceRNA) theory indicated that RNA transcriptions included LncRNAs communication through a new manner mediated by microRNA response elements (MREs).^13^ Here, we listed some LncRNAs functioned as ceRNAs, including oncogenic LncRNAs and tumour‐suppressor LncRNAs.

#### 2.1.1 Oncogenic LncRNAs

LncRNA histocompatibility leucocyte antigen complex P5 (HCP5), which is transcriptional regulated by SMAD3 in NSCLC cells, contains miR‐203 response elements. MiR‐203 expression was down‐regulated in NSCLC and negatively correlated with clinical tumour‐node‐metastasis (TNM) stages.^14^ Jiang et al demonstrated that HCP5 repressed miR‐203 acting as a molecular sponge, stabilized Snail and Slug, and in turn, promoted the invasion of lung cancer cells.^15^ Taken together, HCP5 up‐regulated the expression of Snail and Slug by sponging miR‐203 and activated the TGF‐β/SMAD signal pathway promoting metastasis of NSCLC.

LINC01436 is a long intergenic non‐coding RNA located at chromosome 21q22.12, and its high expression is significantly associated with poor overall survival of patients with NSCLC. Yuan et al found that abnormally high expression of LINC01436 promoted metastasis of NSCLC. LINC01436 functioned as a ceRNA via competitively binding miR‐30a‐3p and regulated target gene endothelial PAS domain‐containing protein 1 (EPAS1).^16^

KCNQ1OT1, known as potassium voltage‐gated channel subfamily Q member 1 (Kcnq1) overlapping transcript 1 or Kcnq1 opposite strand/antisense transcript 1, is an antisense LncRNA. Recently, increasing evidence suggested that KCNQ1OT1 played an important role in tumorigenesis and metastasis of different cancers.^17^ Dong et al revealed KCNQ1OT1 promoted NSCLC progression partly via sponging miR‐27b‐3p in order to target 3′‐untranslated region (3′‐UTR) of HSP90AA1 directly.^18^

ZNFX1 antisense RNA 1 (ZFAS1), a LncRNA, originally identified a regulator of mammary development.^19^ Tian et al discovered that ZFAS1 was up‐regulated in NSCLC tissues and higher expression in more advanced tumour tissues.^20^ It was demonstrated that ZFAS1 exerted as ceRNA to enhance the expression of proliferation, invasion and metastasis‐related genes, such as ZEB1, MMP‐14, MMP16, BMI1, Sp1 and ZEB2 by competitively sponging miR‐150, miR‐200b or miR‐200c.^21^, ^22^

HMMR‐AS1, as a carcinogen, is involved in advanced TNM staging, greater tumour volume and positive lymph node metastasis because of its high expression. Cai et al found HMMR‐AS1 functioned as a ceRNA of miR‐138, and the high expression of miR‐138 caused the repression of its endogenous target SIRT6.^23^ SIRT6 is a direct target of miR‐138, and knockdown of SIRT6 in NSCLC cells could increase the paclitaxel sensitivity.^24^ HMMR‐AS1 could be considered as a potential target for the diagnosis and treatment of NSCLC.

SUMO1P3, located at human chromosome 1q23.2, is firstly identified for its high expression in gastric cancer tissues.^25^ Zhang et al confirmed SUMO1P3 expression was significantly increased in NSCLC cancer tissues and cell lines. Meanwhile, the expression level of SUMO1P3 in metastatic lymph node specimens was up‐regulated in comparison with primary NSCLC tissue specimens.^26^ They also found SUMO1P3 promoted NSCLC cell migration and invasion by repressing miR‐136,^26^ which directly targeted Smad2 and Smad3 to inhibit epithelial‐mesenchymal transition (EMT) process in lung cancer cells.^27^

LncRNA H19 was the first identified LncRNA in 1990.^28^ Although it was reported that H19 was frequently overexpressed in lung cancer and related to cell proliferation,^29^ it was still unclear whether H19 had other function in lung cancer. Recently, Zhao et al discovered that H19 overexpression promoted lung cancer migration and invasion. Mechanistically, H19 captured miR‐200a to decrease miR‐200a target genes, such as ZEB1 and ZEB2, and thereby promoting EMT, cell migration and invasion.^30^

LncRNA MAF BZIP Transcription Factor G Antisense RNA 1 (MAFG‐AS1) had significantly higher expression in NSCLC than the corresponding normal tissues. But, the mechanism of MAFG‐AS1 in NSCLC progression was still not yet explored. Jia et al uncovered that up‐regulation of MAFG‐AS1 promoted the migration, invasion and EMT of NSCLC cell through serving as a miR‐339‐5p sponge to positively regulate the expression of MMP15.^31^ Additionally, previous studies showed that down‐regulation of MMP15 repressed invasion and metastasis in various cancers.^32^, ^33^

Long non‐coding RNA NNT‐AS1, as a new LncRNA, was transcribed in the opposite direction of nicotinamide nucleotide transhydrogenase (NNT). Shen et al showed that the expression of NNT‐AS1 was up‐regulated in NSCLC tissues and cell lines. High NNT‐AS1 expression was associated with advanced tumour stage and lymph node metastasis of patients with NSCLC.^13^ They also identified NNT‐AS1 could function as a ceRNA by sponging miR‐129‐5p in lung cancer. Other research discovered that miRNA‐129‐5p suppressed lung cancer cell proliferation and invasion through targeting microspherule protein 1, E‐cadherin and vimentin.^34^

In addition to the above LncRNAs, many studies revealed that other oncogenic LncRNAs, which could play their roles as ceRNAs, were associated with the NSCLC TNM stage and had higher expression in NSCLC than normal tissues. In Table 1, we listed some other oncogenic LncRNAs competed with miRNAs as miRNA sponges, thereby regulating the downstream targets to promote NSCLC metastasis.

**Table 1:** Other oncogenic LncRNAs as miRNA sponges promoting metastasis of NSCLC

Oncogenic LncRNAs	Target miRNA	Downstream targets	Reference
LncRNA NR2F2‐AS1	miR‐320b	BMI1	35
LncRNA TTN‐AS1	miR‐4677‐3p	ZEB1	36
LncRNA XLOC_008466	miR‐874	MMP2/XIAP	37
LncRNA CAR10	miR‐203/miR‐30	SNAI	38
LncRNA urothelial carcinoma‐associated 1 (UCA1)	miR‐193a‐3p	ERBB4	39
LncRNA NEAT1	miR‐181a‐5p	HMGB2	40
LncRNA SNHG7	miR‐193b	FAIM2	41
LncRNA X inactivate‐specific transcript (XIST)	miR‐367	ZEB2	42
HOXD antisense growth associated long non‐coding RNA (HOXD‐AS1)	miR‐133b	MMP9	43

#### 2.1.2 Tumour‐suppressor LncRNAs

LncNONHSAT081507.1 (LINC81507) was first identified by Peng et al using Agilent Human LncRNA Array.^44^ Recently, Peng et al found reduced expression of LINC81507 resulted in cell growth, proliferation, migration and EMT in NSCLC cells, whereas ectopic overexpression of LINC81507 resulted in the opposite effects both in vitro and in vivo.^45^ LINC81507 acted as a ceRNA for miR‐199b‐5p through directly binding and interfering miR‐199b‐5p‐mediated regulation of CAV1 to reduce migration and invasion. In conclusion, LINC81507 served as a tumour suppressor gene in NSCLC.

The LncRNA growth arrest‐specific transcript 5 (GAS5), a tumour suppressor gene, was significantly down‐regulated in NSCLC tissues and cell lines, and elevated expression of GAS5 inhibited cell proliferation and induced apoptosis in NSCLC cells.^46^ Dong et al demonstrated that down‐expression of GAS5 obviously induced NSCLC migration and invasion. GAS5 acted as a ceRNA of miR‐205 and down‐regulated miR‐205 to suppress lung cancer progression‐related phenotypes via targeting the PTEN mRNA 3’‐UTR to inhibit its translation.^47^

### 2.2 LncRNAs bind with target proteins

LncRNAs can promote or inhibit the metastasis of NSCLC by directly binding with the target proteins.

#### 2.2.1 Oncogenic LncRNAs

BCYRN1 (brain cytoplasmic RNA 1, also known as BC200), a 200‐nucleotide LncRNA, was found highly expressed in some carcinomas of the breast, cervix, oesophagus, lung, ovary, etc, but normally not detectable in the corresponding normal tissues.^48^ Hu et al found BCYRN1 was the target gene of c‐MYC and could mediate cell migration and invasion in NSCLC via influencing the expressions of MMP9 and MMP13.^49^ In conclusion, BCYRN1 is an oncogene, and the metastasis of NSCLC was increased by c‐MYC–activated BCYRN1 promoting the expressions of MMP9 and MMP13.

LncRNA SBF2 antisense RNA 1 (SBF2‐AS1), a 2708 nt antisense RNA to SBF2, was significantly up‐regulated in NSCLC tissues compared with the corresponding non‐tumour tissues. Lv et al found SBF2‐AS1 was a positive factor to promote the metastasis of NSCLC cells. RNA immunoprecipitation discovered that SBF2‐AS2 could bind with a core component of polycomb repressive complex 2, SUZ12. Other chromatin immunoprecipitation assay demonstrated that, after silencing SBF2‐AS1, the enrichment of SUZ12 and trimethylation of histone 3 lysine 27 decreased at the promoter region of P21.^50^

MUC5B‐AS1, a new long non‐coding antisense transcript for MUC5B, was significantly increased in NSCLC tissues. Many studies have explored the relationships between MUC5B expression and clinicopathological characteristics, which found that overexpression of MUC5B was associated with early post‐operative metastasis and poor overall survival (OS) in patients with NSCLC.^51^ Yuan et al demonstrated that MUC5B‐AS1 was up‐regulated and functioned as an oncogene in NSCLC. MUC5B‐AS1 promoted cell migration and invasion by forming a protective RNA‐RNA complex with MUC5B, thereby increasing MUC5B mRNA expression level in NSCLC.^52^

LINC00852 had a positive regulatory role in the progression, migration, invasion and metastasis of NSCLC cells. S100A9 is a calcium‐binding protein. Studies had revealed that tumour cells could secrete S100A9 to recruit myeloid‐derived suppressor cells (MDSCs), thereby promoting cancer growth and forming a special pre‐metastatic immunosuppressive niche.^53^ Liu et al discovered that LINC00852 targeted S100A9 to activate the MAPK signalling pathway, thereby contributing to the formation of a metastatic microenvironment, enhancing NSCLC cell migration and invasion, and eventually facilitating spinal metastasis.^54^

LINC00511 was a newly identified LncRNA, which was up‐regulated in human breast cancer as an oncogene.^55^ Sun et al demonstrated that LINC00511 was highly up‐regulated in both NSCLC tissues and cell lines. Furthermore, LINC00511‐mediated oncogenic effects were partially through its epigenetically silencing of the p57 expression via directly binding with enhancer of zeste homolog 2 (EZH2).^56^

In addition to these above LncRNAs, there were many other oncogenic LncRNAs, which could play their inducing NSCLC metastasis by indirectly influencing signal pathways transduction via binding with proteins. In Table 2, we listed some other oncogenic LncRNAs and their target proteins.

**Table 2:** Other oncogenic LncRNAs and their target proteins inducing NSCLC metastasis

Oncogenic LncRNAs	Target proteins	Reference
LncRNA HOXA‐AS2	IGF2	57
LncRNA Myocardial infarction‐associated transcript (MIAT)	TDP43	58
LncRNA PXN‐AS1‐L	PXN	59
LncRNA LINC00312	Y‐Box Binding Protein 1 (YBX1)	60
LncRNA LINC00707	CDC42	61
LncRNA MALAT1	TDP43	62

#### 2.2.2 Tumour‐suppressor LncRNAs

SPRY4 intronic transcript 1 (SPRY4‐IT1), derived from an intron of SPRY4 gene,^63^ was also a tumour suppressor gene. Wen et al found that the SPRY4‐IT1 expression was significantly lower whereas EZH2 expression was higher in NSCLC tissues. SPRY4‐IT1 and EZH2 showed a negative interaction in patients with NSCLC. EZH2 may promote the invasion and migration of NSCLC cells by inhibiting the SPRY4‐IT1 expression.^64^

LncRNA NORAD was discovered to exploit a novel mechanism for regulating protein function.^65^, ^66^ Tan et al indicated that LncRNA NORAD was down‐regulated in lung cancers and that NORAD low expression was associated with lymph node metastasis and poor prognosis.^67^ Mechanistically, NORAD exploited its multiple repeated sequences to function as a multivalent platform for binding and sequestering S100P, thereby suppressing the associated pro‐metastatic signalling network of S100P.

LncRNA AK126698, a tumour suppressor in NSCLC progression, was found that its expression was lower in cisplatin‐resistant A549/DDP cells compared with parental A549 cells.^68^ Fu et al also showed that AK126698 was significantly down‐regulated in patients with NSCLC and could remarkably inhibit NSCLC cell migration. Their findings suggested that up‐regulated AK126698 contributed to a decreased expression of FZD8, and that was associated with Wnt/β‐catenin inactivation and subsequent up‐regulation of E‐cadherin.^69^

LncRNA CASC2, as a tumour suppressor, had been discovered in many human tumours. Wang et al reported that CASC2 expression was down‐regulated in NSCLC tissue samples and cells, and significantly associated with lymph node metastasis. Overexpression of CASC2 inhibited the expression of SOX4, which acted as an oncogene and induced EMT process of cancers.^70^

LINC00961 was 1546nt in length and located in chromosome 9. Jiang et al uncovered that LINC00961 was significantly down‐regulated in NSCLC tissues, and decreased LINC00961 predicted poor prognosis for patients with NSCLC. Further experiments demonstrated that LINC00961 could act as a tumour suppressor partially via affecting β‐catenin expression.^71^

### 2.3 LncRNAs participate in the transduction of different signal pathways

Signal pathways play very important role in regulating progression and metastasis of tumour, such as Wnt/β‐catenin, PTEN/AKT and Akt/mTOR. Recent studies confirmed that many LncRNAs could exert their regulating NSCLC metastasis function by influencing the transduction of different signal pathways.

#### 2.3.1 STAT3 signal pathway

LINC01288, located on chromosome 8p12, was first reported by Bian et al as an oncogenic factor for NSCLC.^72^ LINC01288 could increase viability and migration of NSCLC cell lines and enhance xenograft tumour growth and metastasis. Mechanistic study showed that LINC01288 could interact with interleukin‐6 (IL‐6) mRNA and increase its stability. The increased expression and secretion of IL‐6 activated STAT3 signalling and therefore promoted the progression and metastasis of NSCLC.

LncRNA tyrosine kinase non‐receptor 2 antisense RNA 1 (TNK2‐AS1), a potential oncogenic LncRNA, was frequently up‐regulated in NSCLC tissues and cell lines. Angiogenesis is one of the hallmarks for tumorigenesis and prognosis.^73^ Wang et al demonstrated that TNK2‐AS1 promoted NSCLC metastasis and interacted with STAT3 to increase its protein stability by protecting it from proteasome‐mediated degradation. STAT3 could also bind with TNK2‐AS1 promoter to trigger its transcription. The positive feedback loop between STAT3 and TNK2‐AS1 therefore activated STAT3 signal pathway by elevating VEGFA expression to induce angiogenesis.^74^

#### 2.3.2 Wnt/β‐catenin signal pathway

Clinically, Wnt/β‐catenin pathway activation predicts increased risk of tumour recurrence in patients with NSCLC.^75^

MIR31HG was a LncRNA, identified as >2166 nucleotides in length.^76^ Recent studies showed that increased MIR31HG expression increased gefitinib resistance in NSCLC lines through the EGFR/PI3K/AKT signalling pathway.^77^ Zheng et al also discovered MIR31HG could promote the invasion of NSCLC by activating the Wnt/β‐catenin signalling pathway.^78^

Brain cytoplasmic RNA 1 (BCYRN1) was a 200‐nucleotide LncRNA that had been reported to be up‐regulated in many malignant tumours, such as lung cancer, breast cancer and ovary carcinoma.^48^ Wang et al confirmed that BCYRN1 was increased in NSCLC, and its ability to induce proliferation and migration was largely due to up‐regulated expression of cell cycle‐related proteins (CDK4 and cyclin D1) and activation of the Wnt/β‐catenin signalling pathway.^79^

NEAT1 is a novel long non‐coding RNA which considered as a crucial regulator in many kinds of tumours.^80^, ^81^ Sun et al found that the expression of NEAT1 in NSCLC tissues and cell lines was much higher than that in normal controls, and NEAT1 could promote the metastasis of NSCLC cells via activating the Wnt/β‐catenin signalling pathway.^82^

#### 2.3.3 PTEN/AKT signal pathway

PTEN/AKT signal pathway is a kind of classic intracellular transduction pathway, and the abnormal activation of this pathway is also related with the development of diseases, such as tumours, autoimmune diseases and diabetes mellitus.^83^

LncRNA ASAP‐IT1, located in chromosome 8q24.21 in whole length of 1179bp, was initially found in ovarian cancer for its abnormal expression.^84^ Zhang et al demonstrated that the expression of ASAP1‐IT1 was relatively up‐regulated in NSCLC cells and tissues, which could promote the proliferation, invasion and metastasis of NSCLC cells through regulating the PTEN/AKT signal pathway.^85^

Focally amplified lncRNA on chromosome 1 (FAL1), a new identified LncRNA located at 1q21.2, had been demonstrated to be up‐regulated and to promote development in several kinds of tumours, especially ovarian cancer.^86^ Pan et al reported that FAL1 was obviously overexpressed in NSCLC tissues compared with the adjacent normal tissues and promoted metastasis of NSCLC via the PTEN/AKT axis.^87^

#### 2.3.4 Other signal pathways

GHET1, a long non‐coding RNA, was found to act as an oncogene in some kinds of tumours. For example, GHET1 promoted gastric carcinoma cell proliferation through increasing c‐Myc mRNA stability, and knockdown of GHET1 inhibited cell proliferation and invasion of colorectal cancer.^88^, ^89^ Guan et al found the expression of GHET1 was increased in NSCLC tissues compared with adjacent normal tissues, and knockdown of GHET1 suppressed cell proliferation and invasion. Moreover, they also demonstrated that knockdown of GHET1 could repress LATS1/YAP signalling pathway by decreasing YAP1 expression in NSCLC cells.^90^

LncRNA small nucleolar RNA host gene 1 (SNHG1) was found overexpressed in diverse cancers, such as liver carcinoma, prostate cancer and NSCLC.^91^, ^92^, ^93^ Zhang et al discovered that SNHG1 could promote ZEB1 protein expression by inhibiting the expression of TAp63, which was one of the isoforms of p63.^94^ It confirmed that SNHG1 might induce the NSCLC metastasis through activating the TAp63/ZEB1 signalling pathway.

MetaLnc9, as a metastasis‐related LncRNA, was identified to be obviously up‐regulated in highly metastatic cells and NSCLC tumour tissues, and its expression correlated with distant metastasis and TNM. Yu et al indicated that MetaLnc9 could interact with the glycolytic kinase PGK1 and prevent its ubiquitination in NSCLC cells, resulting in activation of the oncogenic AKT/mTOR signalling pathway.^95^

NF‐κB interacting LncRNA (NKILA), encoded by a gene at chromosome 20q13 just near by the prostate transmembrane protein androgen induced 1 (PMEPA1),^96^ was increased by NF‐κB in breast cancer.^97^ Recently, Lu et al discovered that NKILA expression was negatively associated with tumour metastasis in patients with NSCLC, and the expression of NKILA was regulated through classical TGF‐β signal pathway, which subsequently inhibited migration and invasion of NSCLC cells through interfering NF‐κB/Snail signal pathway in NSCLC cells.^98^

FER1L4, a novel LncRNA, was first identified that its down‐regulated expression in human gastric cancer.^99^ Gao et al also found that FER1L4 was down‐regulated in NSCLC in vivo and in vitro, and overexpression of FER1L4 could inhibit cell metastasis through regulating the PI3K/Akt signal pathway.^100^ Many studies indicated that the PI3K/Akt signalling was aberrantly activated in human malignancies and was associated with tumour metastasis and drug resistance.^101^

## 3 MAIN REGULATING MANNER OF LncRNAS TO INFLUENCE THE METASTASIS OF NSCLC

### 3.1 Regulate the progression of epithelial‐mesenchymal transition (EMT)

The EMT is closely related to the high invasiveness and metastasis of cancer cells, including NSCLC.^102^ More and more evidence indicates that LncRNAs are involved in tumour invasion and metastasis by regulating EMT.

LncRNA HOX transcript antisense RNA (HOTAIR) exhibited significantly higher expression in the tumour tissues than the adjacent non‐tumour tissues in patients with NSCLC.^103^ In lung cancer cells, HOTAIR was required for the expression of matrix metalloproteinases that break down the extracellular matrix to pave the path.^104^, ^105^ Taken together, HOTAIR was induced by EMT stimuli, and such an induction in turn promoted the gene expression programme that resulted in EMT.^106^ HOTAIR also could potentially regulate lung cancer metastasis through physical interactions with E3 ubiquitin ligases and their corresponding substrates.^106^

LncRNA activated by TGF‐β (ATB) was first identified in liver cancer.^107^ Many studies showed that ATB promoted malignancy in many kinds of cancers, including breast cancer, glioma and colon cancer.^108^, ^109^, ^110^ Recently, Wei et al demonstrated that ATB expression was significantly increased in NSCLC tissues and cell lines, compared with normal controls.^111^ Knockdown of ATB in NSCLC cell lines inhibited the metastasis of lung cancer cells. They also found that suppression of ATB increased the expression of E‐cadherin, while decreasing the expression of N‐cadherin, suggesting that ATB could influence the migratory ability of NSCLC by EMT.

Besides, many other oncogenic LncRNAs were also confirmed to promote the metastasis of NSCLC by inducing EMT, such as LncRNA colon cancer‐associated transcript 1 (CCAT1), LncRNA HNF1A‐antisense 1 (HNF1A‐AS1) and LncRNA LINC00460.

### 3.2 Influence the properties of cancer stem cell (CSC)

Cancer stem cells (CSCs) play an important role in maintaining capacity of tumour metastasis, invasion and recurrence.^112^ Because CSCs contain the self‐renewal abilities which are similar to the normal stem cells, targeting CSCs also can become an effective strategy to treat cancers.^113^ Recently, some LncRNAs are discovered relating with the NSCLC metastasis via influencing the properties of CSCs.

DGCR5, also known as Linc00037, was a kind of LncRNA involved in lung cancer, Huntington's disease neurodegeneration and hepatocellular carcinoma.^114^, ^115^, ^116^ Wang et al reported DGCR5 acted as an oncogene and revealed the possible mechanism for an interaction between DGCR and miR‐330‐5p in lung CSCs. They exhibited that DGCR5 silence could inhibit CSC‐like phenotypes in NSLCL by sponging miR‐330‐5p and increasing CD44 expression.^117^

Linc00662, a newly discovered LncRNA, had a strong correlation with lower overall survival rate and higher lymph node metastasis rate of patients with lung cancer. Lin28, as an RNA‐binding protein and a reprogramming factor, can promote the tumourigenesis and progression in many human cancers.^118^ Evidence indicated that Lin28 also could participate in CSC regulation.^119^ Recently, Gong et al demonstrated that Linc00662 not only induced cell migration and invasive ability but also elevated the CSC percentage in NSCLC cells by the interaction with its downstream factor Lin28.^120^

## 4 CONCLUSION AND PERSPECTIVES

LncRNAs have become a new therapeutic target for inhibiting NSCLC metastasis. Up to now, there are more and more LncRNAs been discovered relating with NSCLC metastasis. In brief, the number of oncogenic LncRNAs is much more than tumour‐suppressor LncRNAs. In this review, we only have summarized the main molecular mechanism and regulating manner of some representative LncRNAs to influence metastasis of NSCLC. Besides the LncRNAs mentioned in this review, there are many other LncRNAs associated with the NSCLC metastasis, such as prostate cancer‐associated transcript 1 (PCAT‐1), linc01433, LncRNA HEIH, LncRNA Homo sapiens TatD DNase domain containing 1 (TATDN1) and LncRNA CASC9.5. But, the molecular basis is still unknown. So it is necessary to do some further research.

**Figure 1:**
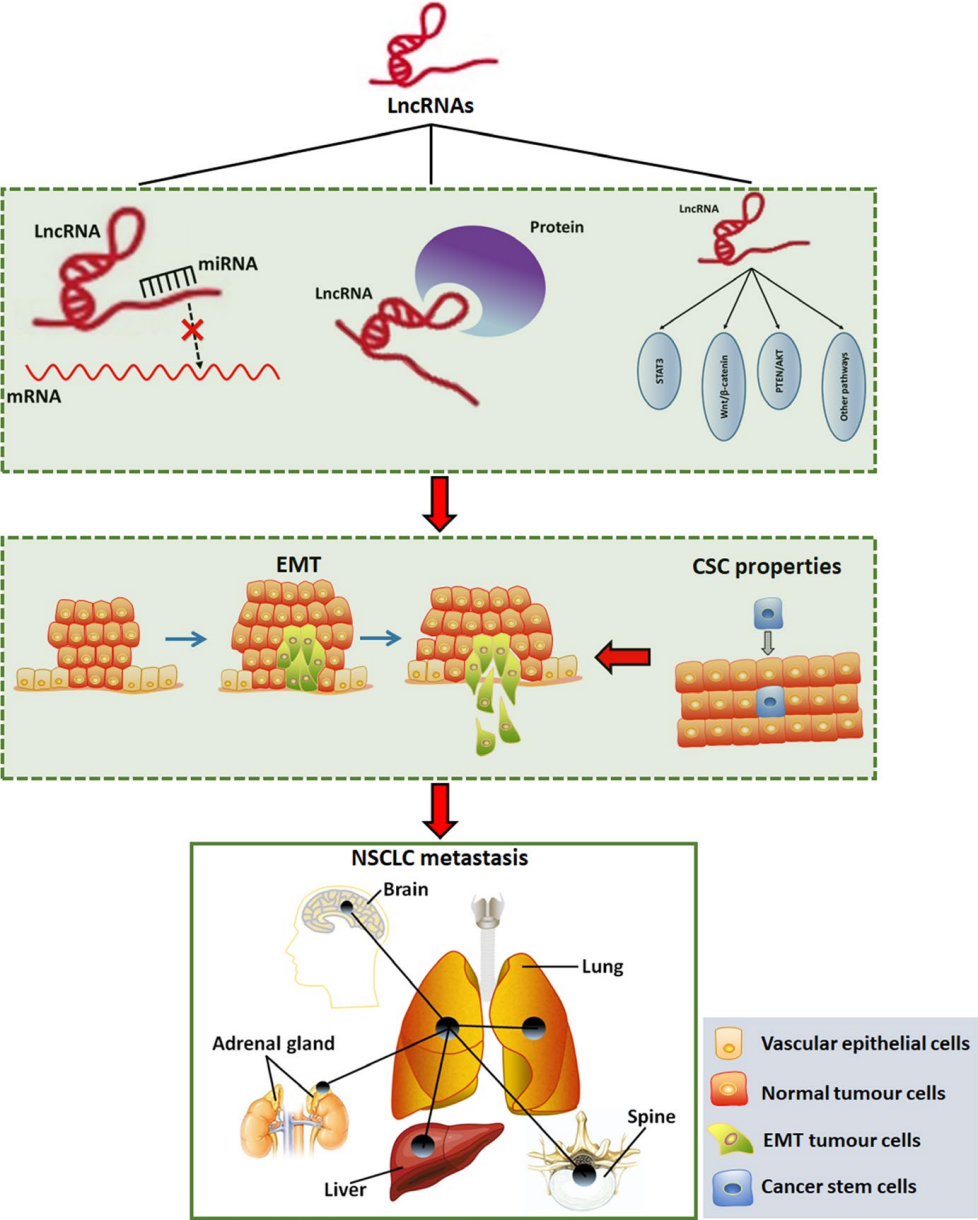
The main mechanism of different LncRNAs to regulate NSCLC metastasis. There are three main mechanism, including: (a) LncRNAs interact with miRNAs as ceRNAs; (b) LncRNAs bind with target proteins; and (c) LncRNAs participate in the transduction of different signal pathways. Then, LncRNAs can exert their function to regulate the metastasis of NSCLC through influencing the progression of EMT and the properties of CSC

According to our review, the molecular mechanism of LncRNAs regulating NSCLC metastasis mainly includes three aspects (Figure 1): (a) LncRNAs interact with miRNAs as ceRNAs; (b) LncRNAs bind with target proteins; and (c) LncRNAs participate in the transduction of different signal pathways. Then, LncRNAs can exert their function to regulate the metastasis of NSCLC through influencing the progression of EMT and the properties of CSC. But as to one LncRNA, the molecular mechanism may contain many aspects. For example, LncRNA‐MALAT1, which is highly conserved among mammals, could not only competitively bind with miR‐145‐5p,^121^ miR‐204^122^ and miR‐206^123^ as a ceRNA, but also activate Akt/mTOR signalling pathway in order to induce the metastasis of NSCLC.^123^ The same LncRNA may have different molecular mechanism in diverse tumours. But until now, the studies in LncRNAs are only at the experimental level. Therefore, ongoing and future studies are expected to clarify the LncRNAs particular regulatory mechanism of inhibiting the metastasis of NSCLC and explore new drugs targeting LncRNAs.

## CONFLICT OF INTEREST

All authors declare that they have no conflict interests.

## AUTHOR CONTRIBUTIONS

Lixin Wang and Shiguo Zhu conceived and designed the manuscript. Cheng Fang, Lixin Wang and Chenyuan Gong drafted the manuscript. Wenbin Wu collected literature. Shiguo Zhu and Chao Yao contributed to the revised version of the manuscript. All authors confirmed the final version of the manuscript for submission.

## Data Availability

I confirm that I have included a citation for available data in my references section.
